# Fine-Grained Assessment of Children’s Text Comprehension Skills

**DOI:** 10.3389/fpsyg.2019.01313

**Published:** 2019-06-28

**Authors:** Marije den Ouden, Jos Keuning, Theo Eggen

**Affiliations:** ^1^Cito, Arnhem, Netherlands; ^2^Faculty of Behavioural, Management and Social Sciences, University of Twente, Enschede, Netherlands

**Keywords:** computer-based assessment, design principles, dynamic assessment, instructional needs, learning potential, reading process, text comprehension

## Abstract

Text comprehension is an essential skill for achievement in personal, academic, and professional life. Therefore, it is tremendously important that children’s text comprehension skills are actively monitored from an early stage. Text comprehension is, however, a complex process in which different reading abilities continuously interact with each other on the word, sentence, and text levels. In educational practice, various tests are used to measure these different reading abilities in isolation, which makes it very difficult to understand why a child scores high or low on a specific reading test and to adequately tailor reading instruction to the child’s needs. Dynamic assessment has the potential to offer insights and guidance to teachers as cognitive processes that are important for learning are examined. In dynamic tests, students receive mediation through instruction when answering test questions. Although computer-based dynamic assessment in the reading domain holds potential, there is almost no support for the validity of dynamic measures of text comprehension. The aim of the present study is to determine design principles for the intended use of computer-based dynamic assessment of text comprehension. Based on the dynamic assessment literature, we developed a model for assessing the different reading abilities in conjunction. The assumption is that this model gives a fine-grained view of children’s strengths and weaknesses in text comprehension and provides detailed information on children’s instructional needs. The model was applied in a computer-based (fourth-grade) reading assessment and evaluated in practice through a three-group experimental design. We examined whether it is possible to (1) measure different aspects of the reading process in conjunction in order to obtain a full understanding of children’s text comprehension skills, (2) measure children’s learning potential in text comprehension, and (3) provide information on their instructional needs. The results show that while the model helped in explaining the children’s text comprehension scores, unexpectedly, mediation did not clearly lead to progress in text comprehension. Based on the outcomes, we substantiate design principles for computer-based dynamic assessment of text comprehension.

## Introduction

Text comprehension is an important skill for personal fulfillment and for achieving academic and professional success. Nevertheless, it is also a very complex skill involving different cognitive abilities that interact on different levels. At lower levels, word identification skills and knowledge of word meanings are essential for understanding text (Perfetti and Hart, 2001; Perfetti, 2017). At higher levels, text comprehension is influenced by the ability to make inferences or monitor comprehension (Perfetti et al., 2005). This complex nature results in a variety of possible causes underlying problems encountered in text comprehension (Cain and Oakhill, 2006; Colenbrander et al., 2016; Kleinsz et al., 2017). Whereas most primary school teachers underline the importance of developing good text comprehension skills, they also point to difficulties understanding the reading problems children encounter. We aim to develop a framework for fine-grained assessment of text comprehension skills that supports teachers in understanding children’s text comprehension problems.

### Measuring Text Comprehension Skills

**Figure 1:**
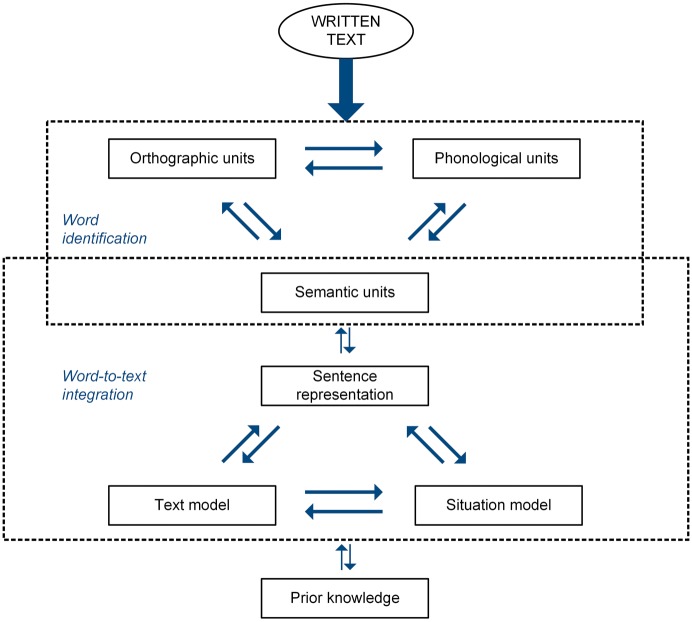
Process of text comprehension, as modeled by Verhoeven and Perfetti (2008).

Research on text comprehension has advanced a number of theories on the different parts of the reading process. Due to the complex nature of text comprehension, interactive models of the reading process arguably provide the best framework for understanding and studying this concept (Stanovich, 1980; Perfetti, 1999; Cain et al., 2017). These models have in common that they describe the reading process of interaction on different levels (e.g., word, sentence, and text levels) and often make a distinction between processing information explicitly stated in the text and deriving information implicitly stated in the text. One of the most influential models is the construction-integration model (Van Dijk and Kintsch, 1983; Kintsch, 1988, 1998), which describes the reciprocal relation between the *construction* of a text-based model and its *integration* into a situation model. A distinction is made between combining all information that is explicitly stated in the text on the word, sentence, and text levels (text model) and interpreting this information, together with prior knowledge, as a coherent whole (situation model). Verhoeven and Perfetti (2008) place greater emphasis on the role of word knowledge by conceptualizing text comprehension as an interaction between word identification and word-to-text integration (see Figure 1). Words are identified by combining orthographic, phonological, and semantic representations. The quality of these representations significantly influences text comprehension (Perfetti and Hart, 2001). Identified words can be linked to each other in order to give meaning to a sentence, and sentences can be linked through inferences based on explicit (text model) and implicit (situation model) information.

In order to obtain a full understanding of children’s text comprehension skills, educational assessment should cover the various aspects of the reading process. In educational practice, a variety of tests are used to measure these different aspects. For example, nationally standardized tests (NSTs) are deployed for student monitoring, i.e., monitoring students’ progress on skills as text comprehension, vocabulary, and word decoding. Additionally, tests originating from teaching materials are administered to evaluate knowledge acquired through education. Consequently, different aspects of the reading process are evaluated through different reading tests, and even tests that are supposed to measure the same construct show only modest intercorrelation (Nation and Snowling, 1997; Keenan et al., 2008). This fragmentary way of measuring reading ability problematizes the interpretation of the test results in a coherent way. Therefore, this way of measuring makes it very difficult to understand why a child scores high or low on a specific reading test and to adequately tailor reading instruction to the child’s needs. Moreover, measuring different aspects of the reading process in isolation is questionable in terms of its interactive nature. It might also be difficult to eliminate every aspect other than that intended for measurement. For example, poor vocabulary can result in an underestimation of inference-making skills (Segers and Verhoeven, 2016; Daugaard et al., 2017; Swart et al., 2017). These issues could be addressed by measuring text comprehension in a more comprehensive way, i.e., measuring different aspects of the reading process in conjunction.

Furthermore, commonly used tests usually provide insufficient diagnostic information, e.g., information about students’ misconceptions and learning potential (Fani and Rashtchi, 2015). Thus, these tests provide only little support for teachers in aligning their reading instruction to the educational needs of their students. *Dynamic assessment* has the potential to offer insights and guidance to teachers as cognitive processes that are important for learning are examined (Lidz and Elliott, 2000; Elliott, 2003). In dynamic tests, students receive mediation through instruction when answering test questions. Dynamic assessment of text comprehension skills can provide teachers with information to identify students’ capabilities as well as their specific needs for training in the reading domain (Dörfler et al., 2017).

### Dynamic Assessment

The idea of dynamic assessment is based on Vygotsky’s (1978) theory of the zone of proximal development (ZPD), wherein human abilities are perceived in a constant state of flux and are sensitive to sources of mediation that can feed learning mechanisms. Lantolf and Poehner (2007) describe two approaches to dynamic assessment: the interactionist and interventionist approaches. The interactionist approach involves the traditional dynamic assessments, whereby the type and amount of instruction provided depend on one-on-one interaction between the teacher and student. The instruction is completely attuned to the responsiveness of the student (Lantolf and Poehner, 2007). In the interactionist approach, the goal is to reach the maximum performance for each individual student. By contrast, the interventionist approach involves standardized instruction that is arranged in advance and quantified during the assessment. This approach focuses on determining the amount and nature of instruction a student needs in order to reach a pre-specified performance level. An interventionist dynamic assessment is less time-consuming, and its results are more comparable across students, since every student is tested according to the same procedure. It enhances efficiency in terms of the number of students that can be tested simultaneously, especially when the assessment is digitalized (Poehner and Lantolf, 2013).

**Figure 2:**
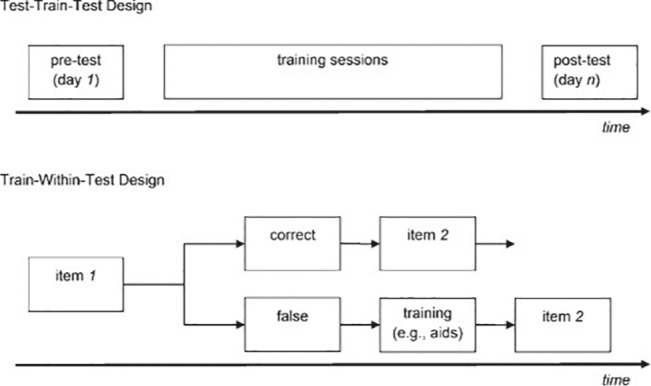
Traditional dynamic assessment design. A test-train-test design (above) with pre-, posttest, and separate training sessions and a train-within-test design (below) with only one session, including training parts (Dörfler et al., 2009).

Computer-based interventionist dynamic assessment can be elaborated through different designs. Sternberg and Grigorenko (2002) distinguish between the sandwich and cake designs. The sandwich design can be defined as a test-train-test design in which a pretest is followed by some intervention or instruction (see Figure 2), and a posttest comparable to the pretest is subsequently administered to all students. With this design, one can determine the extent to which students are able to improve when instruction is offered (Tzuriel, 2000). Performance before and after this instruction can be compared in order to examine students’ ZPD or their potential to learn. The cake design can be defined as a train-within-test design in which instruction follows immediately after an incorrect response to an item (see Figure 2). The instruction can be presented as a graded series of instructional hints that guide the student toward the correct response, referred to as the graduated prompts approach (Brown and Ferrara, 1985; Campione and Brown, 1987). This approach determines the amount of aid a student needs to solve the problem (Tzuriel, 2000). The number of hints needed to find the correct response is often used as an indication of students’ ZPD or learning potential.

### Model for Fine-Grained Assessment

**Figure 3:**
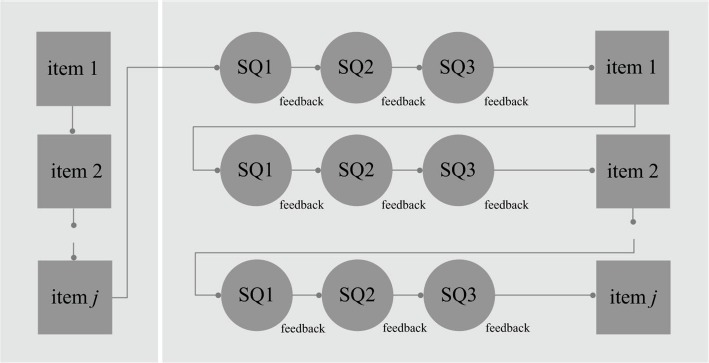
Assessment model for dynamic reading assessment. Performance on the items of the posttest (right squares) can be compared to performance on the items of the pretest (left squares). The change in performance can then be linked to/explained by performance on the scaffolding questions (SQ).

Measuring text comprehension in a comprehensive and dynamic way given the discussed purpose holds some challenges. First, a test of this nature should provide a full understanding of students’ text comprehension skills. All parts and interactions of the reading process should ideally be examined in conjunction. Using the model of Verhoeven and Perfetti (2008), these can be summarized in the constructs word-form knowledge (orthographic and phonological representations), word-meaning knowledge (semantic representations), local cohesion inferences (word-to-text integration), and global understanding (text and situation model). Moreover, this test should inform teachers about the educational needs of their students as well as of the efficacy of intervention. Furthermore, the administration of this test should be feasible. It should take a limited amount of time and ought to be clearly beneficial to both the teacher and student. All these considerations have been accounted for in the assessment model presented in Figure 3, which presents an amalgamation of the sandwich and cake designs.

Both the sandwich and cake designs show some difficulties when used for dynamic reading assessment. In the cake design, quantifying the amount of instruction students need in order to find correct responses can provide an indication of their learning potential; however, it does not allow for modeling the effect of instruction. In the sandwich design, change in performance level caused by the training can be modeled. However, this overall effect cannot be linked to specific types of instruction, since there is only one intervention phase. Multiple training sessions and posttests can address this issue but would nonetheless be highly time-consuming. By combining the sandwich and cake designs, the overall effect of instruction (i.e., learning potential) can be determined and can also be linked to the amount and nature of the instruction offered.

Following the proposed assessment model, a test with the same set of items measuring global understanding is presented in two respective measurement occasions. At the first measurement occasion, a set of items is presented without instruction, and at the second measurement occasion, a set of items is presented with item-level instructions. The instructions consist of several supportive scaffolding questions related to word-form knowledge, word-meaning knowledge, and local cohesion inferences, along with corresponding feedback. At the second measurement occasion, children are thus trained in successfully completing the global text comprehension task by first teaching them the necessary knowledge at word- and sentence level.

### Scaffolding and Feedback

As discussed earlier, dynamic assessment is characterized by the inclusion of instruction during test administration. In this way, dynamic tests provide information about the educational needs of students as well as possible intervention. Research has showed that students with similar initial abilities can benefit differentially from instruction (Tzuriel, 2000). Moreover, different shortcomings in the process of reading might require different approaches from teachers in providing guidance and instruction (Fuchs et al., 2012). In the proposed design, the instruction phase consists of scaffolding questions and feedback on the responses to these questions.

Scaffolding can be defined as providing cognitive support by breaking down tasks into smaller, more manageable parts that are within the student’s understanding (Dennen and Burner, 2008). In the case of reading comprehension, determining the main idea of the text is a cognitively demanding task that can be broken down into smaller tasks as determining (the meaning of) important words and making required inferences between sentences or paragraphs. According to Vygotsky’s (1978) theory of ZPD, students can achieve their potential level of development if scaffolding is applied to them (Magno, 2010), which can be applied in the form of questions, as recommended by Feng (2009). By using a series of scaffolding questions that focus on different cognitive abilities on different levels of the reading process, we can gradually guide a student toward global understanding of a text. Also, we can determine the extent to which a student is capable of making necessary intermediate steps for gaining global understanding of the text. Moreover, different aspects of the reading process can be measured in this way.

Feedback on students’ responses to scaffolding questions is essential for letting them acquire the intended knowledge. Item-based feedback can be presented as either verification or elaboration. Elaborated feedback is more effective than verification; however, they are most effective when combined (Dörfler et al., 2009; Van der Kleij et al., 2015). Verification feedback simply consists of a confirmation of an (in)correct response. Elaborated feedback could contain error-specific explanations and solution-oriented prompts or could address meta-cognitive processes. In the proposed design, standardized solution-oriented prompts are preferable, since non-contingent feedback has been shown to be more predictive of future achievement than contingent feedback in dynamic assessment (Caffrey et al., 2008).

### The Present Study

Although computer-based dynamic assessment in the reading domain holds potential, there are only a few approaches to dynamic assessment available, and thus, there is almost no support for the validity of dynamic measures of text comprehension (Dörfler et al., 2017). The aim of the present study is to determine design principles for the intended use of computer-based dynamic assessment of text comprehension. The proposed, theoretically based assessment model was applied in a computer-based dynamic assessment for text comprehension and tested and evaluated in practice. We examined whether it is possible to (1) measure different aspects of the reading process in conjunction in order to obtain a full understanding of children’s text comprehension skills, (2) measure children’s learning potential in text comprehension, and (3) provide information on their instructional needs. Learning potential was defined as the difference between two measurements occasions, one in which a global understanding task was administered without scaffolding and one in which the same task was administered in combination with several supportive scaffolding questions related to word-form knowledge, word-meaning knowledge, and local cohesion inferences. In this study, learning potential thus reflected the child’s ability to use the help they get in completing the global understanding task. Instructional needs referred to the children’s performance on the different scaffolding questions. Failure on one specific subskill implied that there was a need for additional instruction on that subskill. Based on the conclusions, we substantiate design principles for computer-based dynamic assessment of text comprehension.

## Materials and Methods

### Participants

The study was conducted in cooperation with a school consortium of which four schools participated with their fourth-grade students. Three schools participated with one school class, and one school participated with two school classes. The schools were located in neighborhoods with average and above-average scores in income, employment, and education level, in comparison with the national standard (The Netherlands Institute for Social Research, 2016). The pretest was administered to 169 fourth-grade students aged approximately 10–11 years old. From the pre- to posttest, one school class consisting of 29 students dropped out.

### Materials

#### Texts

A total of 80 texts were selected from a database managed by Cito Institute for Educational Measurement. The database contained texts from existing sources, e.g., children’s books, informative books, and websites. Texts were evaluated by T-scan, an analysis tool for Dutch texts to assess the complexity of the text (Pander Maat et al., 2014). The selected texts were found to be appropriate in terms of difficulty following an evaluation of different text attributes, e.g., word difficulty, sentence complexity, verbiage, and referential and causal coherence. Both informative and narrative texts were included. The selected texts contained between 112 and 295 words, averaging 205 words.

#### Tasks

For every text, four tasks were constructed and screened by a group of reading experts. All tasks corresponding to one text were constructed by one reading expert, screened by two other reading experts and, when necessary, adjusted by the first reading expert. The tasks covered different parts of the reading process, as modeled by Verhoeven and Perfetti (2008), as they represented the constructs *word-form knowledge, word-meaning knowledge, local cohesion inferences*, and *global understanding*.

The different tasks were separately pre-examined in a trial with paper-based tests. Each test consisted of 40 tasks that measured the same construct. Each test was administered to at least two school classes, which resulted in 40–97 administrations per test with a total of 629 administrations. In this preliminary research, all tasks were found to be highly reliable and appropriate with respect to level of difficulty. The resulting item bank consisted of 80 texts and corresponding tasks and was used for the assembly of the final test. Item statistics (i.e., percentage of correct answers and item-total correlation) were used for the test assembly so as to ensure item quality and to maximize task reliability. Texts with too hard (percentage correct < 0.35) or too easy (percentage correct > 0.90) items, or items with a low item-total correlation (<0.20), were not included in the final test.

**Figure 4:**
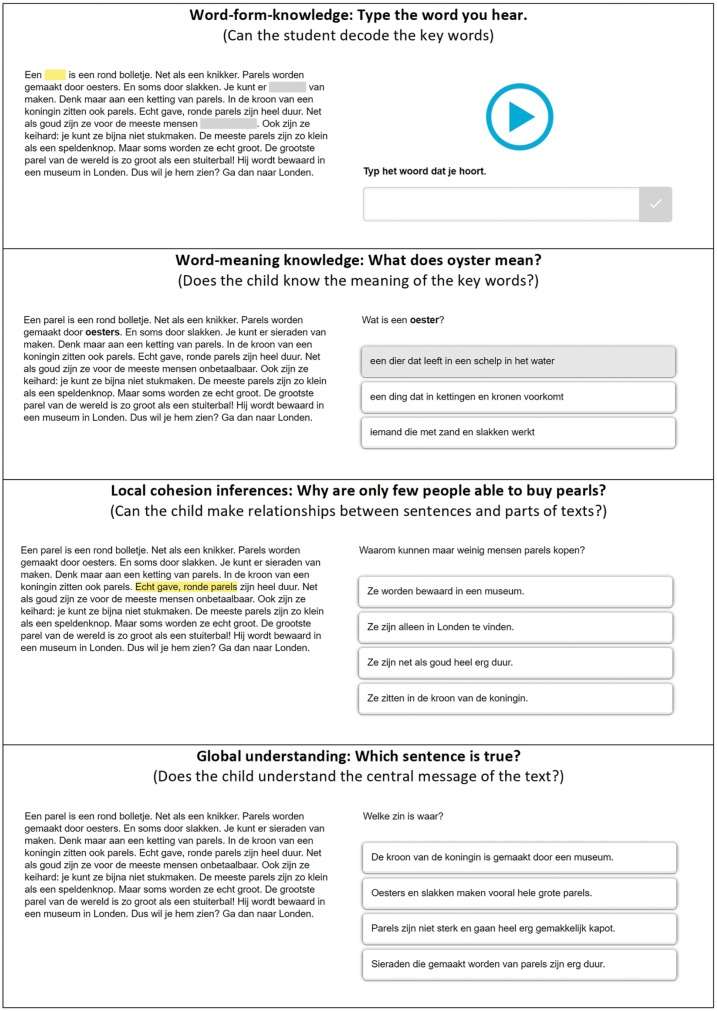
Examples of the four tasks regarding (from above to below) word-form knowledge (SQ1), word-meaning knowledge (SQ2), local cohesion inferences (SQ3), and global understanding.

The final test consisted of 30 texts and was administered twice, as displayed by the squares in Figure 3. During a pretest, each text was presented with one task regarding global understanding of the text. During a posttest, each text was presented with up to four tasks; one task regarding global understanding of the text preceded by, depending on the experimental condition, up to three scaffolding questions with feedback. An example of the tasks is shown in Figure 4.

##### Global Understanding

For every text, the students were asked about the main idea of the text in a multiple choice question with four possible choices. This task measured the ability to integrate all the information provided by the text into a situation model. During the pretest, children had to derive this information from the text themselves. During the posttest, children could use the acquired knowledge from the preceded scaffolding and feedback as guidance for finding the correct response.

##### Word-Form Knowledge (SQ1)

For every text, children were asked to type in three words in three separate open-ended questions. The words were blurred in the text and presented to the students auditory (see upper part of Figure 4). As feedback on an incorrect response, the correct word form was shown in the text for 3 s. This task measured the quality of phonological and orthographical representations of words that were essential for understanding the text. By applying scaffolding and feedback on word-form knowledge, the children could get acquainted with the key words of the text.

##### Word-Meaning Knowledge (SQ2)

For every text, the students were asked for the meaning of two words in two separate multiple choice questions, each with three possible choices of word definitions. The word in question was bolded in the text. The feedback on an incorrect response included a picture of the word in question. This task measured the quality of the semantic representations of words that were essential for understanding the text. By applying scaffolding and feedback on word-meaning knowledge, children received information about the meaning of the key words of the text.

##### Local Cohesion Inferences (SQ3)

For every text, the students were asked to make an inference, relevant for understanding the main idea of the text, in one multiple choice question with four possible choices. As feedback on an incorrect response, the relevant phrases or sentences were highlighted in yellow in the text. This task measured the ability to integrate text phrases that were essential for understanding the text. By applying scaffolding and feedback on inference-making, the children were encouraged to think about the cohesion of different text parts.

#### Procedure

The pretest was divided into two subtests, each with 15 texts that were administered on separate occasions on the same day. The posttest was divided into three subtests, each with 10 texts that were administered on separate occasions spread over two consecutive days. All test administrations took place in the classroom, with a duration of 45 min for each occasion. The posttest was administered 4 weeks after the pretest.

All groups received the same pretest. For the posttest, all students were randomly assigned, within the school classes, to one of three conditions. The first experimental condition (*n* = 47) received the posttest that included all three different types of scaffolding and feedback for every text, SQ1, SQ2, and SQ3. The second experimental condition (*n* = 48) received the posttest that included two different types of scaffolding and feedback for every text, SQ1 and SQ2. The control condition received the posttest that included no scaffolding or feedback (*n* = 45). To ensure active processing of feedback, the students had a second attempt at the scaffolding questions following an incorrect response.

#### Statistical Analyses

In order to determine to which extent we were able to measure different aspects of the reading process in conjunction, the psychometric quality (i.e., reliability and validity) of the developed test was investigated. Classical test and item analyses were conducted for all scales. Internal consistency was assessed with Cronbach’s alpha (*α*), a lower-bound estimate of reliability, with a value of ≥0.80 indicating good reliability, a value of ≥0.70 indicating sufficient reliability, and a value of <0.70 indicating insufficient reliability (Evers et al., 2010).

Furthermore, construct validity was evaluated through the analysis of a multitrait-multimethod matrix (MTMM; Campbell and Fiske, 1959). For this MTMM, scores on the dynamic assessment scales were linked to previously obtained scores on NSTs for text comprehension, vocabulary, orthography, and math. These tests were administered 4 months earlier with the purpose of monitoring students’ progress through primary school. Pearson correlation (*r*) between the scores on the subscales of the dynamic assessment and NSTs was computed and interpreted as high when *r* ≥ 0.50, moderate when *r* ≥ 0.30, and low when *r* < 0.30 (Cohen, 1988).

In order to determine to which extent we were able to measure children’s learning potential in text comprehension and to provide information on their instructional needs, we investigated learning potential and the effect of scaffolding and feedback on *global understanding*. First, the experimental conditions were compared to the control condition on the posttest performance after controlling for pretest performance through a regression analysis. Second, posttest performance was predicted through performance on the scaffolding types and the contribution of feedback.

## Results

### Psychometric Quality

#### Reliability

**Figure 5:**
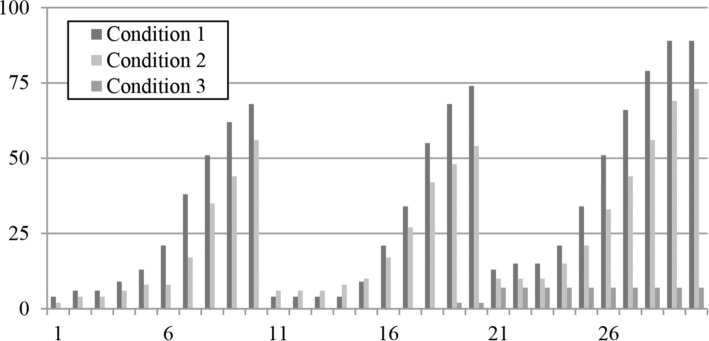
Percentage of missing values per item from the posttest.

In Table 1, the 30 *global understanding* items from the pretest together show good reliability (*α* = 0.82). The same items from the posttest showed even better reliability in the second experimental and control conditions (both *α* = 0.89). However, in the first experimental condition, these items showed very low reliability (*α* = 0.36). An overview of the missing percentage values per item on the posttest are shown in Figure 5. For every subtest, the missing percentage values in both experimental conditions increased considerably as the test continued, indicating that the test was excessively long in these conditions; a large proportion of the children were not able to finish the subtests.

**Table 1:** Reliability of the scale of global understanding.

	*n* items	*n* persons	*α*	90% CI	*μ* rit	*μ p*
Pretest	30	169	0.82	(0.79, 0.85)	0.39	0.60
Posttest						
Condition 1	30	47	0.36	(0.11, 0.56)	0.37	0.49
Condition 2	30	48	0.89	(0.85, 0.92)	0.49	0.52
Condition 3	30	45	0.89	(0.85, 0.93)	0.49	0.65
Shortened pretest	18	169	0.74	(0.69, 0.79)	0.41	0.60
Shortened posttest						
Condition 1	18	47	0.73	(0.62, 0.81)	0.43	0.56
Condition 2	18	48	0.85	(0.80, 0.90)	0.51	0.54
Condition 3	18	45	0.83	(0.76, 0.88)	0.50	0.65

When the observations for the last four items of every subtest were excluded from the analyses, Cronbach’s alpha for the *global understanding* scale exceeded 0.70 in the first experimental condition (*α* = 0.73) and decreased only slightly in the second experimental and control conditions (*α* = 0.85 and *α* = 0.83). Thus, in the first experimental condition, the observations made for the last few items of every subtest showed a negative effect on the reliability of the total scale. Therefore, we chose to proceed with all analyses with only the items corresponding to the six texts presented at the beginning of every post-subtest, leaving a total of 18 texts. As presented in Table 2, the corresponding 54 *word-form knowledge* items together showed good reliability in both experimental conditions (*α* = 0.90 and *α* = 0.91) as well as the 36 *word-meaning knowledge* items (*α* = 0.79 and *α* = 0.88). The 18 *local cohesion inference* items, which were only administered in the first experimental condition, together showed poor reliability (*α* = 0.47). Therefore, we cannot make any statements about the children’s ability to make local cohesion inferences.

**Table 2:** Reliability of the scaffolding scales.

	*n* items	*n* persons	*α*	90% CI	*μ* rit	*μ p*
Word-form knowledge						
Condition 1	54	47	0.90	(0.86, 0.93)	0.31	0.60
Condition 2	54	48	0.92	(0.89, 0.95)	0.39	0.60
Word-meaning knowledge						
Condition 1	36	47	0.79	(0.72, 0.86)	0.30	0.73
Condition 2	36	48	0.88	(0.83, 0.92)	0.40	0.71
Local cohesion inferences						
Condition 1	18	47	0.47	(0.27, 0.64)	–	–

#### Validity

The multitrait-multimethod matrix (MTMM) for the dynamic assessment scales and NSTs is presented in Table 3. The scale *global understanding* shows a high correlation with the NST that measures the similar construct of text comprehension (*r* = 0.51) as well as the NST measuring construct vocabulary (*r* = 0.50). The scale *word-meaning knowledge* shows a high correlation with the NST that measures the similar construct of vocabulary (*r* = 0.52) and a slightly higher correlation with the NST measuring the construct of text comprehension (*r* = 0.59). The scale *word-form knowledge* shows a high correlation with the NST that measures the similar construct of orthography (*r* = 0.83) and lower correlations with the NSTs measuring less related constructs. Furthermore, from the intercorrelations between the subscales, we can conclude that *word-form knowledge* discriminates better with *global understanding* and *word-meaning knowledge* (*r* = 0.47 and *r* = 0.52) than the latter do among themselves (*r* = 0.68).

**Table 3:** Multitrait-multimethod matrix for the dynamic assessment scales and nationally standardized tests.

		Dynamic assessment (DA)		
Method	Trait	Global understanding	Word-meaning knowledge	Word-form knowledge
DA	Global understanding	(0.88)		
	Word-meaning knowledge	0.68	(0.91)	
	Word-form knowledge	0.47	0.52	(0.93)
NST	Text comprehension	**0.51**	0.59	0.50
	Vocabulary	0.50	**0.52**	0.46
	Orthography	0.34	0.46	**0.81**
	Mathematics	0.33	0.31	0.32

The dynamic assessment scales are based on the posttest data for Conditions 1 and 2 together. The values in parentheses represent the reliability (*α*) of the scale. The values of the validity diagonal are shown in bold and are expected to be high. All correlations were found to be statistically significant different from zero (*p* < 0.05).

### Learning Potential and Instructional Needs

To determine children’s learning potential, posttest performance on *global understanding* was predicted through the conditions after controlling for pretest performance. Compared to the control condition, both experimental conditions showed a negative effect on posttest performance, indicating that scaffolding deteriorated posttest performance (see Table 4).

**Table 4:** Regression coefficients predicting posttest performance controlled for pretest performance.

	Unstandardized coefficients		Standardized coefficients	
	*B*	SE	*β*	*p*
Pretest	0.637	0.073	0.589	<0.001
Condition 1^*^	−2.074	0.673	−0.242	0.002
Condition 2^*^	−2.186	0.668	−0.256	0.001

R^2^ = 0.387, F(3, 136) = 28.61, p < 0.001.

* Condition 3 served as the reference category.

To determine whether we were still able to provide information about children’s instructional needs, posttest performance on *global understanding* was predicted by performance on the scaffolding tasks and the contribution of feedback. Scaffolding was operationalized as a percentage of the items that were answered correctly during the first attempt. Feedback was operationalized as a percentage of the items that were answered incorrectly during the first attempt and correctly during the second attempt. Since both experimental conditions received word-level scaffolding and feedback, we chose to include both groups in the same model, with the condition as a control variable and the first condition as the reference category. The predictors explained a significant part of the variation in posttest performance, *R*^2^ = 0.477, *F*(5, 89) = 16.25, *p* < 0.001. From the results shown in Table 5, we can conclude that scaffolding on both *word-form knowledge* (*β* = 0.209, *p* = 0.073) and *word-meaning knowledge* (*β* = 0.784, *p* < 0.001) was a relevant predictor of global understanding. The feedback showed no significant contribution. Although no significant effects could be proved, the high standardized beta for feedback on *word-meaning knowledge* suggests the potential relevance of this type of feedback (*β* = 0.212, *p* = 0.240).

**Table 5:** Regression coefficients predicting posttest performance on global understanding.

	Unstandardized coefficients		Standardized coefficients	
	*B*	SE	*β*	*p*
Word-form knowledge				
Scaffolding	4.752	2.619	0.209	0.073
Feedback	3.844	4.488	0.090	0.394
Word-meaning knowledge				
Scaffolding	19.256	4.635	0.784	<0.001
Feedback	8.049	6.801	0.212	0.240
Control variables				
Condition 2^*^	0.094	0.602	0.012	0.877

Scaffolding was operationalized as a percentage of the items that were answered correctly during the first attempt. Feedback was operationalized as a percentage of the items that were answered incorrectly during the first attempt and correctly during the second attempt.

* Condition 1 served as the reference category.

Since the experimental conditions did not perform better on *global understanding* than the control condition, children’s learning potential could not be assessed. Within the experimental conditions, however, scaffolding proved to be relevant for explaining performance on *global understanding*. Therefore, we were able to provide diagnostic information on children’s text comprehension skills.

## Discussion

In order to define design principles for fine-grained assessment of text comprehension skills, a computer-based dynamic assessment based on the proposed assessment model was developed and evaluated in an experimental design. We examined to what extent we were able to measure a combination of the different aspects of the reading process by evaluating the quality of all scales. We found that a large proportion of the children in both experimental conditions were unable to finish the subtests of the posttest, indicating that these tests were excessively long. In relation to *global understanding*, the test length showed a negative effect on the reliability of the scale in the experimental condition, where children received both word- and sentence-level scaffolding and feedback. Thus, in particular, scaffolding and feedback on the sentence level (i.e., *local cohesion inferences*) resulted in inconsistent response behavior on the *global understanding* scale, indicating concentration and motivation challenges. The inclusion of six texts per subtest proved to be the maximum for obtaining a reliable *global understanding* scale. Proceeding with the analyses with only these texts, w*ord-form knowledge* and *word-meaning knowledge* were also evaluated to be reliable scales.

The *local cohesion inferences* scale was found to be highly unreliable on the computer-based dynamic assessment, though it was found to be perfectly reliable and well-constructed when administered in isolation with a paper-based test in the preliminary research. Two possible explanations are conceivable for this difference. First, these items could function differently on a computer-based test than on a paper-based test. To find the correct response to the items, it is necessary to read the text and find the relevant text phrases. Reading a text presented on a computer screen usually entails higher cognitive workload than reading a text presented on paper (Mangen et al., 2013). Second, the items could function differently when administered in isolation, compared to when they are administered together in a series of tasks. For every text, the children were first presented with the items regarding their word knowledge. In order to find the correct responses to these items, reading the text could have helped, though it was not necessary. Finding the correct response to the *local cohesion inferences* item, however, required the children to use information from the text. Moreover, making inferences is perceived as a higher-level ability and is, therefore, more cognitively demanding than activating word knowledge, which is perceived as a lower-level ability. The change in the required approach to problem-solving might have caused confusion or motivational problems.

We, therefore, concluded that the underlying constructs measured by the scales *global understanding* and *word-meaning knowledge* overlapped considerably. Also, both scales showed almost equal coherence with other tests that measured text comprehension and vocabulary in isolation. This could be explained by the essential role of vocabulary in text comprehension as well as by the inability of the other tests to measure text comprehension and vocabulary as separate abilities, since these abilities continuously interact and influence each other (Verhoeven et al., 2011; Oakhill and Cain, 2012). Correlations of 0.80 between tests for reading comprehension and vocabulary are not uncommon (e.g., Tomesen et al., 2017, 2018), and this supports the assumption that different reading abilities should be measured in conjunction.

To determine whether we were able to measure learning potential, the posttest performance on *global understanding* was compared for the experimental conditions versus the control condition after controlling for the pretest performance. On average, those children who received scaffolding and feedback were found to perform slightly worse than those who received no scaffolding or feedback. This could be variously explained.

As discussed earlier, children might benefit differently from instruction. Therefore, linking learning potential to specific characteristics might provide more meaningful information, since it allows for the identification of groups with similar educational needs. However, the sample size of the present study was too small to determine learning potential for smaller groups. A larger sample size would also allow for the estimation of test scores with the use of item-response theory models. These models can provide more accurate scores, as they take into account the difference between the difficulty of an item and the ability level of a student.

The most likely explanation for the lack of finding a positive effect of scaffolding and feedback concerns the possible incomparability between pre- and posttest performance on *global understanding* as well as between the experimental and control groups. When the *global understanding* task is integrated in a series of tasks, the conditions under which the children perform change. The required change in approach to problem solving between the different tasks, as pointed out earlier with respect to the *local cohesion inferences* scale, can affect children’s performance or the difficulty of the tasks. Shifting the focus from measuring learning potential to measuring instructional needs would provide teachers with more valuable information.

Another probable explanation is that the information retrieved from the scaffolding and feedback was not used for the *global understanding* task. The children did not receive explicit information about the structure of the test; consequently, they themselves had to realize that they could use the previously collected information to solve the task. Moreover, previous research suggests that computer-delivered elaborated feedback is likely to be neglected in a low-stakes assessment setting on higher-order processes of text comprehension (Golke et al., 2015). Motivating children to use the information provided might address this problem.

To determine whether we were able to provide information on children’s instructional needs, performance on *global understanding* was predicted by performance on scaffolding and the contribution of feedback. Scaffolding on *word-form knowledge* and *word-meaning knowledge* proved to be relevant for *global understanding*. The contribution of feedback on *word-meaning knowledge* could not be proved, although there were indications that it might be proved in a larger sample. Previous research has indicated the efficacy of using pictures when learning new words (Gruhn et al., 2019, under review). Therefore, further research is necessary to determine the contribution of this type of feedback within the assessment model. Feedback on *word-form knowledge* showed no contribution to the prediction of *global understanding*. This might be due to the lack of repetition (Gruhn et al., 2019).

Thus, some important information on children’s instructional needs could be provided. However, further research is required, since inference-making skills could not be reliably assessed, and being able to integrate multiple sentences is essential for achieving *global understanding* of a text (Best et al., 2005).

## Conclusion

Based on the findings we can conclude that the assessment model can be used as framework for fine-grained assessment of text comprehension skills when some design principles are taken into account. First, children should be informed about the test structure in advance. In this way, they can be explicitly instructed to use information retrieved from the scaffolding or feedback. Second, (sub)tests should be relatively short so as to avoid fatigue effects that cause biased results. We recommend a maximum of six short (ca. 200 words) texts per subtest. Third, a pretest where one’s ability is measured in isolation might not be a good baseline for establishing learning potential. Further research is required, however, since the inability to establish learning potential in the present study might have had other causes. In any case, comparability of response behavior elicited by pre- and posttest measures should be examined beforehand. Fourth, the negative effects of changes in the required approach to problem-solving or the cognitive workload between different tasks should be diminished. These effects might be reduced by presenting a visual indication of the level of difficulty or a sign reflecting the task type of every task. In the present study, the change from the *word-meaning knowledge* task to the *local cohesion inference* task seems to cause problems in particular. In addition to a visual indicator or (warning) sign, reducing the cognitive workload for this specific task may also contribute. This could be achieved by adding a new, in-between task that serves as an extra intermediate step or by directly highlighting the relevant text passages instead of only after an incorrect response. However, attention must be paid to the influence of such adjustments on the validity of the task itself and the following tasks.

To conclude, we have tried to bridge the first gap between theory and assessment by evaluating the theoretically based assessment model for fine-grained assessment of text comprehension skills in practice. We were able to measure a combination of different aspects of the reading process. Furthermore, we suggested that it might be more valuable to focus on instructional needs rather than on learning potential. Through the design principles discussed, we can move further toward fine-grained assessment of text comprehension skills.

## Ethics Statement

We obtained written informed consent from all participating schools and the children’s parents were informed about the study by letter. The parents had the opportunity to refuse participation of their child in the study. Active written informed parental consent was not considered necessary because we only collected non-special personal data; biographical information or any other private or health-related data were not part of our study. This particular sub-study was conducted under a research grant of the Netherlands Initiative for Education Research (NWO/NRO dossiernummer 405-15-548), which was approved by a scientific and ethical review committee. Therefore, because of institutional (Cito) and national (NOW/NRO) guidelines and regulations, the separate approval by an Ethics Committee was not required.

## Author’s Note

The following data were collected in our study: (1) Correct/incorrect scores on a dynamic assessment for text comprehension and (2) test scores for reading comprehension, spelling, vocabulary and math from a Dutch Student Monitoring System.

## Author Contributions

MO is a PhD student. JK is the daily supervisor. TE is the supervisor of the project. The authors are equally responsible for the content.

### Conflict of Interest Statement

TE and JK were employed by Cito in The Netherlands. They are affiliated with Stichting Cito (Foundation Cito), the not-for-profit part of Cito that is dedicated to applied scientific research into educational measurement. MO was employed at the University of Twente.
